# The “sphere of care”: A qualitative study of colorectal cancer patient and caregiver experiences of support within the cancer treatment setting

**DOI:** 10.1371/journal.pone.0209436

**Published:** 2018-12-26

**Authors:** Eleanor Law, Janelle V. Levesque, Sylvie Lambert, Afaf Girgis

**Affiliations:** 1 ACT Health, Canberra, Australia; 2 Centre for Oncology Education and Research Translation (CONCERT), Ingham Institute for Applied Medical Research, Liverpool, Australia; 3 South Western Sydney Clinical School, UNSW Medicine, The University of New South Wales, Liverpool, Australia; 4 Ingram School of Nursing, McGill University, Montreal, Canada; Maynooth University National University of Ireland Maynooth, IRELAND

## Abstract

**Introduction:**

Colorectal cancer is associated with considerable physical and psychosocial burden. Whilst social support is known to facilitate psychological adjustment to cancer, patients’ and caregivers’ experiences of social support within a treatment setting and their perceptions of the role of the treating team in providing this support is unknown. Specifically, there is a gap in the research that explores in detail who people affected by colorectal cancer consider to be supportive, and the function, timing and nature of this support, whilst receiving treatment. This study explored both patients’ and caregivers’ a) experiences of social support and how this relates to their experience of treatment; and b) what facilitates support in the treatment setting.

**Methods:**

Individual interviews (N = 20) were conducted with patients diagnosed with colorectal cancer and caregivers of such patients. Audiotaped interviews were transcribed verbatim and analysed using the framework method.

**Results:**

Three major themes emerged from the data: a) treating team as a source of support, highlighting the importance of connection with the treating team; b) changes in existing social supports, encompassing issues regarding distance in interpersonal relationships as a consequence of cancer; and c) differing dimensions of support, exploring the significance of shared experience, practical, financial, and emotional support.

**Conclusions:**

Patients and caregivers perceived the treating team as a major source of support. Support from the treating team was particularly important in the context of the changes that occur as a result of a diagnosis of colorectal cancer and the effects of subsequent treatment. Incidental support from others encountered in the treatment setting was also experienced and was equally important to both patients and caregivers. This has implications for the way health care professionals respond to both patients and caregivers in the treatment setting in terms of communication, interventions and environment.

## Introduction

It has long been acknowledged that social support is an important function of social relationships and psychological health [1, 2]. Social support is of significant importance for individuals affected by cancer, as they often experience high levels of personal stress due to the changes in daily living, work, relationships and roles that are experienced as a result of cancer [3]. Social support can help alleviate the associated stress for patients [4], as well as for caregivers. Higher levels of caregiver burden have been associated with lower perceived social support [5].

Whilst the volume of research on the relationship between cancer and social support has grown substantially over the past two decades, with an increase in the number of well-conducted sufficiently powered studies much of this research has taken place in the breast cancer population [6–8]. Published evidence for other cancer s reporting on social support following diagnosis and treatment for cancer is sparse, and overall the results are variable. Breast cancer studies suggest support declines [9–12], although one study reported stability [8]. There are limited studies from other cancer sites; a study in prostate cancer found that social support remained stable [13] whilst another found support declined for head and neck cancer patients [14]. Patients with CRC and their caregivers have been largely understudied in terms of their experiences of social support [15–17], with a limited number of studies reporting that social support declines over time [16, 17]. For example, results from the Colorectal Wellbeing (CREW) study [15], a longitudinal study following more than 1,000 people with CRC from before their surgery until five years afterwards, reported changes in social support following diagnosis, identifying emotional support as critical, with those reporting a lack of emotional support more than three times as likely to experience poor well-being [15]. The psychological impact of colorectal cancer (CRC) is significant for some, with a recent systematic review reporting higher prevalence of depression (19 versus 12.8 percent) and anxiety (20.9 versus 11.8 percent) for CRC patients as compared to population samples [18]. Social support has been identified as helpful, and appears to be somewhat protective in terms of mental health outcomes for patients with CRC [19]. For example, in a qualitative CRC study by Ó Céilleachair, Costello [20], social support from family and friends was found to contribute considerably to patient well-being.

To date, the social support experience for patients with CRC and their caregivers within the cancer treatment setting had largely been unexplored. Specifically, there is a gap in the research that explores in detail who people affected by CRC consider to be supportive, and the function, timing and nature of this support, whilst receiving treatment.

Understanding the role of such support in alleviating stress associated with a cancer diagnosis and treatment is of interest, as it may offer some explanation into differential psychosocial outcomes for people affected by CRC. The treatment setting is important as, for many people affected by cancer, treatment can take place over many months, sometimes years, and encounters in this setting are potentially a form of social support. Additionally, evidence suggests that over time, social support from friends and family may wane [21], which may elevate the importance of support received within the treatment setting and its potential impact on psychosocial adjustment and well-being. Furthermore, research that incorporates caregivers is required as the role of social support for this population is not well understood. The research aim of this study was to gain an in depth understanding of CRC patients’ and caregivers’ experience of social support within the cancer treatment setting.

## Methods and materials

### Study design

Ethical approval was obtained to conduct this qualitative study from the ACT Human Research Ethics Committee (ETH. 11.14.317). Written consent was gained from participants prior to the participating in the study. The qualitative methodology was chosen, as it allows for an examination of people’s lived experiences and behaviours, and the attributed meaning for them as individuals [22].

### Participants

Participants were recruited from the Radiation Oncology Department at The Canberra Hospital, Canberra, Australia. Participant inclusion criteria were: a) diagnosis of CRC or caregiver for a patient with CRC, b) aged 18 years and over, c) sufficiently fluent in English to be able to complete the interview, d) cognitively able to comprehend the implications of consent and participate in an interview and e) having finished or having cared for someone who completed radiotherapy treatment for CRC within the last four months (for two patients chemotherapy treatment was ongoing). Time since diagnosis was not incorporated in the eligibility criteria, as the focus of the study was on social support relative to their recent treatment experience. Thirty patients and caregivers (16 patients/14 caregivers) who had completed radiation treatment were initially identified by health professional staff as being eligible for the study. Seven were did not meet the timeframe of having finished treatment within the previous 4 months and were not approached. Thus 23 patients or caregivers who were approached face to face by a cancer nurse specialist to take part in the study, one person declined and two others were not included, as they were unable to complete the interview within a four week period. Twenty participants took part in the study, 12 patients and eight caregivers. Initially they were recruited as consecutive patients/caregivers who had recently completed treatment. After the first nine interviews, purposeful sampling was employed to ensure the range of participants recruited reflected the whole population of people affected by CRC. Recruitment ceased when data saturation was established as evidenced by no new themes or insights emerging in the data collected [23]. Demographic and time since diagnosis data were also collected.

### Data collection

Semi-structured 1:1 interviews were conducted with all participants individually. Sixteen face-to-face interviews were conducted in private rooms at The Canberra Hospital, Australian Capital Territory, Australia; three interviews were completed over the phone and one interview was conducted at the participants’ home at their request. All interviews were conducted by the first author (EL, a female registered psychologist with eight years of clinical experience in psycho-oncology and an interest in the role of social support in cancer settings). Participants were unknown to the interviewer, interviewed once, and flexible use of the interview guide was employed with respect to the order of questions and the depth of exploration of some issues. No other persons were present during the interview and participants were informed of the basis of the research and signed consent forms prior to commencement. Field notes were made following the interviews, which ranged from 11 to 62 minutes (M = 29 minutes, SD = 14 minutes). All interviews were audio-recorded and transcribed verbatim, with transcription performed by an external company. No transcripts were returned to participants for feedback.

The interview schedule was developed based on an a priori framework, developed following a review of the literature examining studies investigating the social support experiences of patients and caregivers affected by cancer, with a focus on CRC where studies were evident. The topic guide was influenced by the Stress and Coping theoretical framework [24], specifically the role of social support in influencing appraisal and coping with relation to a CRC diagnosis and treatment. The topic guide covered the impact of cancer, experiences and perceptions of social support, and preferences for support within the treatment setting. A pilot interview with a CRC patient was recorded and transcribed prior to commencement of the study to ensure format and structure of the interview was appropriate, it was not included in the final analysis. The interview guide included prompts (S1 Interview Guide).

### Data analysis

Analysis was conducted using the framework approach, a qualitative content analysis that consists of five interconnected stages that are linked to form a methodical and rigorous audit trail [25–27]. The framework approach can be used for both a deductive and inductive approach and Gale et al. [25] recommend a combined approach. A deductive approach incorporates themes and codes that are pre-selected based on previous literature, previous theories or the specifics of the research question; whereas in the inductive approach, themes are generated from the data though open (unrestricted) coding, followed by refinement of themes. Gale et al. [25] suggest a combined approach is appropriate when the project has some specific issues to explore, but also aims to leave space to discover other unexpected aspects of the participants’ experience or the way they assign meaning to phenomena. This combined approach was adopted for the current study.

One author (EL) coded all transcripts by hand to identify initial themes. Two authors (JL and EL) independently coded the first three transcripts to ensure codes were interpreted consistently and ensure no particular perspectives of the researcher dominated. To ensure rigor, data analysis and discussion of cases and codes were discussed at regular research meetings. Six transcripts were coded independently by the first and second authors (EL and JL), revealing close agreement on the emerging themes. This approach was undertaken to involve the cross checking of coding strategies and interpretation of data by two independent researchers [28]. It also provided insight and discussion that was valuable in refining the coding frames [28]. Five stages of data analysis are involved in the framework approach: familiarisation with all the data, identification of a thematic framework (using a-priori aims and issues and themes expressed by participants), indexing (systematic coding of the data), charting (grouping the data in charts thematically, comparing within and across participants) and mapping and interpretation (exploration of the themes, with relation to overarching patterns and explanation of the findings in relation to the aims of the study) [25, 29]. Participants were not invited to provide feedback on findings. Table 1 outlines an example of application of the analytical framework for the development of themes.

**Table 1:** Application of framework analysis for colorectal patient and caregiver experiences of support within the cancer treatment setting.

Initial framework based on a priori knowledge	Revised framework based on indexing	Themes	Sub-themes
Treating team’s support differs from support received from existing social network (caregiver included in discussions, type of support, delivery setting, acknowledgment of impact of disease)	Perception of treating team with regard to providing support (provision of appropriate support, caregiver role, setting, personalized experience important)	Treating team as a source of support	Understanding of impact of disease; Personalised experience; Perception of treating team’s competence; Acknowledgment of caregiver
Challenges patients and caregivers face in terms of changes brought about by cancer (disruption to lifestyle, managing others’ responses, seeking help, delivery of support)	Social network changes as a result of cancer (changes in multiple facets of life–social, work, etc.; reluctance to ask for help, friends avoiding contact/ unhelpful support, unexpected support)	Changes in existing social support	Cancer changes everything; Reluctance to ask for support; When support lets you down, Stepping up
Differences in support experience outside of the treating team (interaction with others in same situation, practical, financial, emotional support, unhelpful support)	Experiences of support (incidental support, types of support–emotional practical, financial)	Differing dimensions of support	Patient shared experience; Practical support; Emotional support; Financial support

### Maintaining research quality

The reporting of the findings was guided by the Consolidated Criteria for Reporting Qualitative Research [30]. Additional steps to ensure methodological rigour included addressing credibility, goodness and transferability criteria. Credibility was addressed by recording and transcribing interviews [26], regular discussion of findings [31], an exploration of negative cases [32], and by employing flexible use of the interview guide to allow participants’ perspectives to be explored [26]. Goodness was addressed by collecting and managing the data in a clear and transparent manner and by choosing an analysis method appropriate for the data [33]. Transferability was addressed by using direct quotes [34], providing detail on the sample and setting [35] and by relating the findings to other literature [36].

## Results

### Participants

The age of the participants ranged from 31 to 82 years. Eleven of the participants were male and nine female. Fourteen of the participants were married or in a defacto relationship, two were widowed, one was single, and one was divorced/separated. Two same sex couples completed the study.

#### Themes

**Fig 1:**
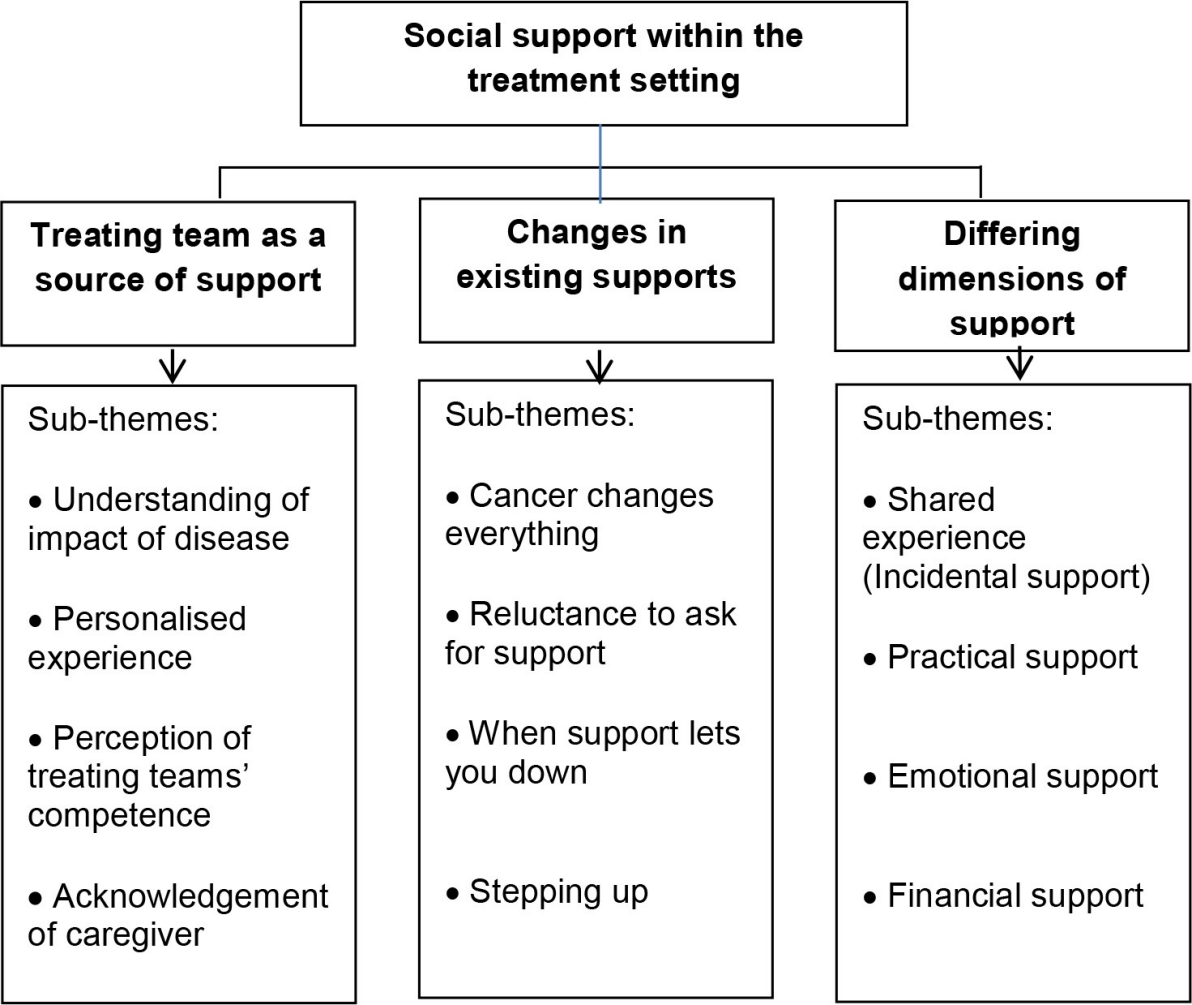
Social support themes and their constituent sub-themes.

A total of three major themes emerged from the data: a) *Treating team as a source of support*; b) *Changes in existing supports*; and c) *Differing dimensions of support* (Fig 1). As the data were analysed it became apparent that the experiences of patients and caregivers were very similar, therefore the data were conceptualised as a coherent set. Participants did not provide feedback on the findings.

#### Treating team as a source of support

The most commonly cited form of social support was represented by the theme *treating team as a source of support*. The treating team incorporated nurses, doctors and allied health staff. This theme was defined as the perception that the treating team cares for and assists patients and caregivers and included four sub-themes: a) *understanding of impact of disease*; b) *personalised experience;* c) *perception of treating team’s competence*; and d) *acknowledgment of the caregiver*.

The subtheme, *understanding the impact of disease*, referred to the participants feeling the treating team understood the burden of disease, whereby they did not need to explain the impact of disease. This support was frequently referred to as multidimensional in nature, *“understanding of the enormity of it and the impact it has on*, *not just that patient*, *but every aspect of your life”* (Sarah, caregiver) and, *“I have to deal with it on all levels*, *not just physically*, *and now that my doctor is saying the same thing*,*…*. *that’s been helpful to me”* (Margie, patient). Health care professionals’ true understanding of the burden of disease was perceived as particularly supportive, with participants reporting they could be honest with the treating team about the personal impact of the disease and treatment, as opposed to family and friends who they were sometimes trying to protect.

*I just felt I was in a kind of—a sphere of care*, *that we’re in good hands and the people were caring about us*, *and that we didn’t have to be brave*.*… So you’re kind of in a different place than when you are with friends and family who are seeing it (cancer) as the threat it is* (Barbara, caregiver).

The subtheme *personalised experience* referred to patients and caregivers highly valuing the treating team adopting a person centred approach. This encompassed care that was provided with an empathic and appropriately responsive approach. Frequently this took the form of taking time to listen, *“even in a short period of time you can make someone feel listened to and supported and I found that they have done that*, *overwhelmingly”* (Jess, patient). Staff members who went ‘beyond the disease’ were also perceived as caring about the individual as a whole, “*that's the priority*, *the primary level of care [treating cancer]*, *but there was also the psychological care there”* (Barbara, caregiver). This frequently incorporated a relaxed, personal manner, which was perceived positively, “*the oncology staff*, *we all know them by first name…*. *they are very positive*, *they’re very supportive and very caring”* (John, caregiver).

A personalised experience led to the report of close ties with the treating team, *“the main support has been my close family and [doctor and nurse care co-ordinator]”* (Jess, patient). For some, this was in contrast to the support received from family and friends. Support from the treating team that was deemed appropriate and sensitive was reported as particularly helpful, and was often preferred to support from friends.

The experience of personalised support was not universal, with three participants commenting on negative staff interactions. These interactions were experienced negatively, mainly because the treating team did not take the time to listen to patients or caregivers.

*She'd [doctor] ask you [a] silly question about something and then you start- before you even finish it*, *“Oh*. *It’s all right—doesn't matter now*,*” and then everything that was going*, *I thought—I just had that sense—why did I even bother coming in here*? *Why would I want to go through this*?. . . . *It’s not right*. (Rob, patient)

The *perception of the treating team’s competence* subtheme referred to having confidence in the treating team and a sense that they provided good care and appropriate, timely advice. The perceived expertise of the treating team was of paramount importance and this in itself was a source of support, as indicated in terms used by participants, such as “*safe hands”* (Sam, patient). Specialisation in particular was highly valued, with sentiments being expressed such as *“I think that the people that we deal with in the cancer centre know cancer”* (Sarah, caregiver). Confidence in the treating team’s ability to take care of patients was particularly important to caregivers, and appeared to be viewed as supportive, “*makes me feel more comfortable knowing that he is getting looked after*. *So I think that’s sort of bit supportive*, *…*.*they’re doing all they can to help him”* (Jane, caregiver).

#### Changes in existing supports

The second theme, *changes in existing supports*, was defined as the changes to existing social networks that occurred as a result of cancer. The four sub-themes that comprised this theme were a) *cancer changes everything*; b) *reluctance to ask for support*; c) *when support lets you down*, and d) *stepping up*.

The sub-theme, *cancer changes everything* referred to the impact cancer has on multiple facets of life. It encompassed diminished access to social supports, dealing with side effects/symptoms, impact on work and energy levels, and isolation for both patients and caregivers. Social isolation brought about by cancer had a considerable impact on both patients’ and caregivers’ lives, *“the longer the treatment goes*, *obviously*, *the more people sort of move on and don’t make contact anymore*, *so that sort of makes you feel a little bit isolated”* (Mark, patient). Sometimes social isolation was directly related to treatment, “*I can't get out much…*. *It's hard when the thing that is the focus of your life is cancer”* (Jess, patient). This was experienced by caregivers as well, especially when needing to relocate for treatment.

*The time available to see people is so diminished …*. *every time we come away from home*, *we nearly always have to stay*.*… otherwise*, *it’s a five-hour return trip …*. *So that means we've got two days or three days away from home and then we go back and we need a day to recover* (Kate, caregiver).

Symptoms and side effects associated with cancer were also reported to impact on social opportunities.

The *reluctance to ask for support* sub-theme was mostly represented by a desire not to burden others, which appeared to be often associated with not wanting to appear vulnerable: “*it’s the things that we shouldn’t–didn’t want to ask for*. *You don't want to say to someone*, *“Can you -*?*”* (Jason, patient), and, *“it’s hard to reach out to other people for support”* (Jo, caregiver).

However, a minority of participants were not reluctant to ask for support and actively made people aware of their situation so they could access support:

*I was really clear because we are isolated that I wasn’t going to get into that situation of not telling anybody…*. *So I was really clear…*. *with [patient] straightaway that I was going to tell everybody*, *that we weren't going to keep it a secret …*.*I needed my employer to be really clear that I was going to need some help sort of with time and flexibility to navigate through all of this*. *So my first thing was*, *right*, *we’re just going to be really open and honest about that and I think that’s proven to be really helpful* (Dexter, caregiver).

The sub-theme,*when support lets you down*, refers to both negative social support experiences which were reported by eight of the 20 participants. This took the form of pre-existing social supports (family and friends) becoming avoidant or providing inappropriate support which was not helpful. Whilst this sub-theme was not present for all participants, it should be noted that it appeared to be upsetting for those who did report it, highlighting that when expected support was not forthcoming, it can be difficult at a time when stress is elevated. For example, “*we’ve actually had people that we thought we’re quite close to us*, *just not be there*. . . . *So it sort of—yeah*, *it’s a very confusing time”* (Sarah, caregiver). For this caregiver this avoidance resulted in irreparable change in a previously close relationship, stating “*as a result of [partner’s] diagnosis*, *they’re sort of not so much there*. *That’s not back to normal*, *that’s sort of broken now*.*”*

Detrimental support, actions and information that were not helpful but well intentioned, were also identified. Patients reported on unhelpful advice provided by well-meaning social supports, “*sometimes friends’ help can actually not be very helpful*. *It’s nice and kind of them to be suggesting pseudo-science bunk*, *but it's also quite damaging”* (Jess, patient). Support not meeting needs was also reported, for example one caregiver (Jane) spoke of receiving lots of support in the form of meals, but stated what she really needed was a cleaner. Sarah (caregiver) expressed her frustration with unmet need, *“Oh*, *if you need anything*,*” it’s just such an empty suggestion…*. *they actually don’t come through with anything that you need*, *when you need it*.*”*

The sub-theme *stepping up* refers to unexpected sources of social support mostly received from friends or community members. This was a common experience, with this unexpected support taking many forms. For example, one patient, a single older man who lived in a rural setting, spoke of members of his community creating a roster, whereby someone would drive him over one hour each way to the hospital for treatment, commenting, *“they were there just to—alongside me*. *That really helps to take my mind off things*.*”* (Arthur, patient)

Contact initiated by social supports was deemed as particularly valuable, “*[When] a friend of yours is actually proactive is really*, *really good because I’ve noticed how important it is for both of us”* (Barbara, caregiver). Reports of help from other patients or caregivers they had never previously met were common and resulted in changes to existing social networks, *“we’re really good friends from that experience”* (Bailey, patient).

#### Differing dimensions of support

The theme, *differing dimensions of support*, encompasses the types of social support participants received from friends, family and HCPs or in some cases desired. The four sub-themes that made up this theme were a) *shared experience*, b) *practical support*, c) *emotional support* and d) *financial support*.

The sub-theme, *shared experience*, refers to support one receives from other patients and caregivers through the shared understanding of similar experiences, with the added benefit of normalising their own experience. In many cases this support was incidental in origin e.g. a chance encounter in the hospital.

Meeting others with a shared understanding gave a sense that you were not going through this alone, and appeared to be in itself supportive, as described Mary (patient), *“Well*, *you always knew that they were all having the same experience…*. *you knew they were going through the same as you”*

Some participants reported that they could be more honest with fellow patients and caregivers as they understood what they were going through, *“so we can talk about things that we probably won't talk to anybody else about”* (Ivan, patient). This shared experience in some way created a bond between strangers and opened the door for supportive connections, even if only briefly. At times, patients adopted a more targeted approach to support, by actively seeking out others who had had the same diagnosis or treatment. Alternatively, for others, support was forthcoming without any prompting, mostly by others who had undergone a similar experience. Having a designated cancer centre appeared to be highly valued from the perspective that participants felt there was a shared understanding of the experience of cancer, as exemplified by Sue (caregiver):

*A good thing having the cancer centre as a centre on its own…. because you do know that everyone’s going through the same, similar thing….. and I’m sure a lot of people feel the same way, that cancer is probably the worst it’s going to get…. in terms of frighteningly you’re going to die. So, they have to make you feel special that here is a special building just for you, and everyone in this building knows what you’re going through, and appreciates that you’re all in the same kind of bucket ….I think that felt very supportive*.

The *practical support* sub-theme refers to assistance received that was more functional in nature, commonly taking the form of provision of meals, transport to appointments and help with household chores. One participant spoke of receiving support of up to seven hours driving in a day to attend appointments interstate, whilst support in the form of meals or help at home was appreciated, as Kate (caregiver) elaborated, “*the boys came down and did… several trailer loads of firewood”*. Practical support in particular appeared to ease the pressure on caregivers who were often trying to juggle multiple demands. When practical support was forthcoming it appeared to send a message of care for the recipient, “*everybody then sort of mobilised themselves*, *so we suddenly got a kind of a wall of willingness and helpfulness”* (Bailey, patient).

Whilst there was a desire for practical support, it wasn’t always forthcoming and when this occurred participants were loathe to ask for help, as reported in the sub-theme *Reluctance to ask for support*.

The *emotional support* subtheme refers to support that assured the individual that they are loved and valued. Many of the participants spoke of the value of regular contact from their pre-established social supports. This often took the form of phone contact or regular emails from friends “*constantly checking in to see how we are going*” (Dexter, caregiver). Support for patients from their pre-established support network appeared to help caregivers as well, *“he opens up and you can see that’s important for us both”* (Barbara, caregiver).

Emotional support was often described as the person listening and taking an interest in what was happening, this could be from friends, family, fellow patients and caregivers or HCPs. One participant spoke of his change in interpretation of support as a result of cancer, *“I've realised that the friends who want to talk about it are wanting to do it*, *not because of some macabre interest in it*, *but because they're interested in my well-being”* (Will, patient). Emotional support provided by health care providers (HCPs) was also frequently reported, “*part of the support network is the community nurses”* (Stuart, patient).

The availability of emotional support was particularly important as a source of support to caregivers, “*one [friend]…*. *was there*, *the whole way through*, *and I was in contact with him and he just listened and kept updated and popped in and*, *you know*, *that was a huge support”* (Sarah, caregiver). In terms of emotional support, it appears it is not the number of supports that are important but the strength of those ties. Patients and caregivers often identified one to two key supports, in some instances these supports were members of the treating team.

The sub-theme *financial support* incorporated monetary support. This sub-theme was a stronger theme for younger participants, which may be indicative of the financial pressures they faced with mortgages, child rearing and disruption to work due to their own or their partner’s cancer.

There was a strong desire for more financial support to assist with the lack of income whilst receiving treatment, as outlined by Jess (patient), “*the financial burden is heavy*. *It's very*, *very heavy*. *I have income protection for two years*, *but that's only two years*. *It's going to run out and I'm going to have cancer for more than two years*.*”* Another patient, Sam, spoke of the cost of treatment and the impact on his finances, “*I’m in a mess at the moment*. *I didn't think this would cost me money*, *but it’s [cancer] cost a lot of money”*. This participant was referring to out of pocket expenses such as diagnostic scans and medications.

While there was evidence of financial hardship, some participants also spoke of significant support with finances. One caregiver reported financial assistance from family members, whilst a younger patient Jess, who had to give up work, had support from friends, *“When I was first diagnosed*, *a friend of mine set up funding*, *a Go Fund Me account*. *And I got $12*,*000 within a couple of weeks to keep me going”*.

## Discussion

This study provides an insight into the social support experiences of CRC patients and caregivers within the treatment setting, an area that has not been well researched to date. The qualitative design allowed for an in depth investigation of the complex and contextual support experience [37]. Whilst qualitative and quantitative studies conducted on cancer patients and their caregivers suggest that HCPs are an important source of support to people affected by cancer [7, 38, 39], this study explored how HCP support fits in the context of all other sources of support, specifically within the CRC patient and caregiver population.

A positive relationship with the health care team was a valuable resource in terms of support for both patients and caregivers. Specifically, this positive relationship appeared to make the treating team approachable for patients and caregivers, facilitating communication and information seeking. An interesting finding to emerge was the importance of both formal and informal communication with the treatment team. Notably, the data highlighted how positive and respectful communication seemed to influence patients’ and caregivers’ perceptions of support.

Whilst physician and health care staff active listening and empathy have been identified as an important element in patient-provider concordance in disease treatment in the broader health care literature [40, 41] there has been limited research directed to colorectal cancer. The current study highlighted that when HCPs’ interaction with CRC patients and caregivers was not perceived as caring, it was damaging to the therapeutic relationship. A recent systematic review of patients’ experiences of communicating with primary care physicians identified negative experiences such as being treated disrespectfully, experiencing pressure due to time constraints, and feeling helpless due to the dominance of biomedical issues [42]. This study extends these findings by exploring a CRC specific population, highlighting that when communication is not delivered in a caring and compassionate manner, it is also viewed as negative. This is important, as previous research in a breast cancer population has found that the deleterious effects of negative interactions to be stronger than the beneficial effects of positive interactions [43].The current study highlights that these findings are also transferable to HCP communication in CRC treatment settings. It may be that communication that is viewed as positive is even more significant within the CRC oncology setting, due to the life threatening nature of cancer, the stigma associated with the disease [44] and the significant psychosocial burden that can be associated with the disease [19, 45]. It appears that in this setting the doctor-patient-caregiver relationship demands a high level of trust and compassion, and as such good communication is imperative.

Although other studies have identified emotional support from HCPs as influential in their adjustment to disease [38, 40, 46], this study accentuated what was valuable in terms of support from the treating team for people affected by CRC as compared to other social supports. The treating team were considered highly competent and their care and advice was considered valuable, whereas informational advice from family and friends was often considered unhelpful and stressful to deal with. This finding is supported by a number of studies that were published some time ago. For example, research by Dunkel-Schetter [47], found that perception of support was dependent on who provided it, in turn contributing to patient stress and adjustment. Similarly, Dakof and Taylor [21] found not all support networks were helpful for cancer patients, and Neuling and Winefield [48] reported informational support from the treating team was desirable, but not from family and friends. The current study updates the literature on this topic, highlighting its relevance for contemporary cancer care. It is possible that this desire for support from the treating team is accentuated in the CRC population due the very private nature of problems experienced as a consequence of the disease. As the participants in this study had all received treatment over a prolonged period, due to the nature of their diagnosis (advanced CRC or rectal carcinoma), it is possible that for this subgroup of patients with CRC, and their caregivers, support from the treating team was particularly valuable by reason of the complex nature of their treatment regime, side-effects and prognosis, as compared to those with earlier stage disease.

Although studies have investigated the impact of the caregiver role from the caregivers’ perspective [3, 49], this study highlighted that acknowledgment of the caregiver role was also highly valued by patients. Previous research has identified a lack of engagement from HCPs with caregivers [50] and caregiver dissatisfaction with their interaction with HCPs [51]. However, the findings in the present study differ, notably that the caregivers were satisfied with the care they received from the treating team, in both the informational and emotional support domains. It is however acknowledged that the current study was conducted in a single location, and therefore the finding may reflect the personnel and culture of patient care at that site. Regardless, this finding has implications for clinical practice, as it demonstrates the importance of HCPs in addressing caregivers’ needs.

The social network changes reported in this study highlighted the impact of cancer on multiple aspects of life, a reluctance to ask for support, and experiences of support. Such themes are present in other cancer research [52, 53]. It is feasible that the stigma associated with CRC may have played a part in social supports not delivering much needed support. A numbers of studies have identified CRC as a taboo subject, impacting on uptake of CRC screening [54–56]. It is possible that the discomfort associated with talking about CRC in particular impacts on access to social support.

For some participants in the study a long or complicated treatment regime left them vulnerable to social isolation. Patients with CRC frequently report that social support declines over time [15, 17]. This has implications for psychosocial health with both CRC caregivers [57] and CRC patients with poor social support reporting higher rates of depression [15]. Therefore, the treating team’s role in providing emotional support for those who have prolonged or complicated treatment regimens for CRC is possibly crucial within the context of pre-existing social support that has undergone change.

Participants in the current study highlighted how they were the recipients of support from unanticipated sources, in the sub-theme of *stepping up*. Breast cancer research lends support to the finding that people affected by cancer frequently receive support from unexpected sources [58, 59]. The current study’s exploration of this form of social support in the CRC patient and caregiver population expands our understanding, as this has not specifically been explored in the CRC patient and caregiver population to date. Specifically, it appears when both patients and caregivers are clear and honest about their needs, they are more likely to receive beneficial outcomes in terms of delivery of social support. However, it should be noted that for the majority of the participants there was a reluctance to ask for support.

The sub-theme, *shared experience* explored the social relationships that emerged as a result of sharing the experience of cancer, specifically within the treatment setting. Most participants spoke of developing relationships with people they had met as a result of cancer, with half referring to receiving treatment in a designated cancer centre as facilitating opportunities for informal social interaction with peers. Previous research has shown that peer support can be very helpful for patients. For example, a systematic review of peer support programs [60] reported a high level of satisfaction with peer-support programs, however, much of the research on peer support has focused on more formal networks, or structured interventions [60, 61]. The current study suggests that informal peer support is also valuable for both CRC patients and caregivers and extends our understanding on its incidence, how incidental support occurs, why it is important, and how it can be facilitated.

The importance of *emotional support* for both patients and caregivers is well established [59, 62, 63]; and our study accentuated the desired amount of emotional support and sources of such support. For those who did actively seek support, their needs were largely met. Furthermore, it seems that the quality of support was important, as even for those with less support reported the support they received as valuable when it was perceived as “appropriate”. This finding is again supported by research in people affected by breast cancer [12] and the current study extends the small literature addressing CRC patient and caregiver populations.

Although the subtheme *financial support* was not one of the stronger themes in the study, it was a significant issue for most of the younger participants, with financial burden most likely exacerbated by their prolonged CRC treatment regimes. Recently, two studies have confirmed the relationship between financial burden and Quality of Life in cancer survivors [64, 65] and financial burden is also commonly reported by cancer caregivers [3, 66]. The findings of this study highlight the financial impact of cancer, and suggest a strong desire for financial support, identifying the types of support that participants identified as beneficial, specifically early intervention in the treatment phase to access benefits, leave from employment, and financial support.

## Study limitations

The findings for this study are limited by the fact that all participants were sampled from a single cancer treatment centre; therefore, the findings may not apply similarly across all CRC patient and caregiver populations. Local organisational factors may have influenced the delivery of care, thereby limiting the transferability of results to different contexts. Whilst the open questioning and inductive approach aimed to reduce the possibility of participants saying what they thought the researcher wanted to hear, participants may have reported their experience of the treatment centre and staff more favourably, since the research itself was conducted at the hospital providing their ongoing care. Whilst not a limitation, it should also be noted that the sample is not representative of all CRC patients and caregivers, as these patients had all undergone radiation therapy, indicating either a diagnosis of rectal carcinoma or more advanced disease. However, the value of this homogeneous sample is that it provides insight into a particularly vulnerable sub-group within CRC who have previously been neglected in the research arena.

## Conclusions

Whilst the majority of participants in this study reported positive treatment experiences, a number of areas were identified where steps could be undertaken to improve their support experience. This study identified that both patients and caregivers appear to be sensitive to interactions with HCPs, valuing a relationship with their treating team that involves sharing of expertise in conjunction with being treated with kindness and compassion. There is also an expectation that caregivers are acknowledged within this process. The significance of this relationship could be addressed within an ongoing communication skills training program for all HCPs with the aim of improving communication of patient centred goals [67], in conjunction with development of pathways to assist with meeting patients’ and caregivers’ information and support needs within their treatment context. In addition, it is recommended that screening for psychosocial issues take place at regular intervals during the treatment trajectory and beyond [68].

Receiving treatment at a designated cancer treatment centre was identified as a supportive environment which facilitated informal supportive interaction with other patients and caregivers. The cancer treatment areas within hospital environments could potentially be purposefully designed to facilitate such “incidental” support; and HCPs could structure waiting room and treatment environments to enhance opportunities for increased interaction between other patients and caregivers. This might include scheduling treatment for people with CRC at similar times, and/or in close proximity to one another, depending on feasibility. Furthermore, programs targeting patients with cancer and their caregivers, such as art therapy [69] or mindfulness classes [70] are encouraged within cancer treatment settings, to increase opportunities for informal interaction with other patients and caregivers. Education of HCPs with regard to engagement with caregivers to explore needs during treatment may also be beneficial with a view to enhance communication and self-efficacy in relation to psychosocial issues [50]. Advice early in the treatment trajectory from allied health and financial services to assist with financial issues is also recommended.

Our findings suggest that HCPs need to be attentive to the complex social experience of people affected by cancer, and CRC more specifically, so they can respond appropriately to patient and caregiver need.

Whilst previous research has focused on support provided by the treating team from a formal perspective, concentrating on the effectiveness of interventions [71] or physician communication style [38], our qualitative findings accentuated what was valuable for people affected by CRC in terms of support from the treating team. Specifically, our participants highlighted that informal interaction from all HCPs is important, as this interaction contributes to a feeling of being supported. Furthermore, when this relationship is fostered, it aids communication and information seeking, which facilitates needs being met. The current study provides an understanding of what makes a treatment experience positive for patients and caregivers which is crucial in the delivery patient-centred care.

Finally, it is recommended that more research addressing CRC specifically is warranted to establish whether the nature of the disease itself is socially isolating, which would contribute further to a comprehensive understanding of the psychosocial impact of CRC.

## Supporting information

S1 Interview Guide (PDF)
