# Supplementary material for: The “sphere of care”: A qualitative study of colorectal cancer patient and caregiver experiences of support within the cancer treatment setting
Source: PLoS One. 2018 Dec 26;13(12):e0209436. doi: 10.1371/journal.pone.0209436 (PMC6306237; doi:10.1371/journal.pone.0209436)
Supplement: S1 Interview Guide — (PDF) [file pone.0209436.s001.pdf]

Interview Guide

1) How did you react to the news that you/the person you are caring for had colorectal cancer? (prompt- People react to the diagnosis of cancer in different ways – some people try to make sense of the situation, others try to find solutions, while others try not to think about it at all as it is too stressful – which of these best describe the way you reacted?)
2) What was most stressful about the diagnosis/ treatment? (prompt – side effects, impact on home life, employment, finances)
3) What has helped you cope with the diagnosis/treatment? (prompt – family support? support from others?)
4) Did you/the person you care for receive treatment have to relocate to local accommodation in order to receive treatment? How has that impacted on the social support you have access to? (prompt – Has your network changed as a result of treatment?)
5) Describe interactions you have had with others at the treatment centre?
    a. If so, with whom? (prompt -other patients, carers or family members, staff, hospital volunteers)
    b. Did you consider them to be supportive? If so, how?
    c. If not, do you think that would have been beneficial?
6) Do you feel the waiting room environment is conducive to meeting others undergoing treatment/ supporting someone through treatment? In what way?
7) Do you feel you have been a source of support to others going through a similar experience to you during this time? If yes, in what form has this been?
8) Explain your relationship with your/ the person you are caring for treating team ? Do you see part of their role as providing personal support? (prompt – What is different about the support you receive form the treating team as opposed to other supports?)
9) Has your social network changed since you stopped treatment? (prompt – Have you seen or do you intend to see any of the patients/other carers you formed friendships with?, Have you resumed other social contacts?)
10) Would you say your experience has changed the way you think about cancer now?
11) Reflecting back on your whole experience of cancer so far, if you could have had access to ANY type of support you wanted, what would that look like? (prompt- before versus after; emotional/practical)
12) As you move past this treatment phase, what support will you like? (prompt – Would you attend a peer support group? Would you be interested in meeting others in the same situation?)
